# Sector-Based Regression Strategies to Reduce Refractive Error-Associated Glaucoma Diagnostic Bias When Using OCT and OCT Angiography

**DOI:** 10.1167/tvst.12.9.10

**Published:** 2023-09-15

**Authors:** Keke Liu, Qi Sheng You, Aiyin Chen, Dongseok Choi, Elizabeth White, Jonathan C. H. Chan, Bonnie N. K. Choy, Kendrick C. Shih, Jasper K. W. Wong, Alex L. K. Ng, Janice J. C. Cheung, Michael Y. Ni, Jimmy S. M. Lai, Gabriel M. Leung, Ian Y. H. Wong, David Huang, Ou Tan

**Affiliations:** 1 Casey Eye Institute, Oregon Health & Science University, Portland, OR, USA; 2 Department of Ophthalmology, Duke University School of Medicine, Durham, NC, USA; 3 Department of Ophthalmology, LKS Faculty of Medicine, The University of Hong Kong, Hong Kong; 4 School of Public Health, LKS Faculty of Medicine, The University of Hong Kong, Hong Kong; 5 The State Key Laboratory of Brain and Cognitive Sciences, The University of Hong Kong, Hong Kong; 6 Healthy High Density Cities Lab, HKUrbanLab, The University of Hong Kong, Hong Kong; 7 Department of Ophthalmology, Hong Kong Sanatorium & Hospital, Hong Kong

**Keywords:** glaucoma, refractive error, OCT, OCTA, false-positive diagnosis

## Abstract

**Purpose:**

This cross-sectional study aimed to investigate the sectoral variance of optical coherence tomography (OCT) and OCT angiography (OCTA) glaucoma diagnostic parameters across eyes with varying degrees of refractive error.

**Methods:**

Healthy participants, including individuals with axial ametropia, enrolled in the Hong Kong FAMILY cohort were imaged using the Avanti/AngioVue OCT/OCTA system. The OCT and OCTA parameters obtained include peripapillary nerve fiber layer thickness (NFLT), peripapillary nerve fiber layer plexus capillary density (NFLP-CD), and macular ganglion cell complex thickness (GCCT). Sectoral measurements of NFLT, NFLP-CD, and GCCT were based on sectors and hemispheres.

**Results:**

A total of 1339 eyes from 791 participants were stratified based on spherical equivalent refraction: high myopia (<−6 D), low myopia (−6 D to −1 D), emmetropia (−1 D to 1 D), and hyperopia (>1 D). Multivariable broken stick regression models, accounting for age, sex, and signal strength, showed that all NFLT sectors except temporally, the inferior GCCT hemisphere, and half of the NFLP-CD sectors were more affected by ametropia-related covariates than the corresponding global parameters. As expected, the false-positive rates in those sectors were elevated. Finally, sector-specific axial length (AL) and spherical equivalent (SE) adjustments helped reduce the elevated false-positive rates.

**Conclusions:**

The effect of optical magnification is even more prominent among sectors than the global parameters. AL- and SE-based adjustments should be individualized to each sector to mitigate this magnification bias effectively.

**Translational Relevance:**

Identifying sectoral differences among diagnostic parameters and adopting these sector-based adjustments into commercial OCT systems will hopefully reduce false-positive rates related to refractive error.

## Introduction

Optical coherence tomography (OCT) is a fast, non-invasive, and reproducible imaging modality that provides a quantitative assessment of structural damage in glaucoma—often before functional changes are detected by perimetry.^1^^,^^2^ Examples of OCT measurements to detect and monitor glaucoma progression include the peripapillary retinal nerve fiber layer thickness (NFLT) and the macular ganglion cell complex thickness (GCCT).^3^^–^^5^ With the advent of OCT angiography (OCTA), a software extension of OCT that separates vasculature motion signals from static ones, pre-perimetric glaucoma can now be evaluated based on the angiographic assessment of retinal layers.^6^^–^^8^ In the peripapillary region, the nerve fiber layer plexus capillary density (NFLP-CD) is one of the best indicators of glaucomatous damage.^9^^,^^10^

Previous studies investigating the sectoral changes in retinal NFLT in eyes with myopia either did not correct the magnification effect or still were correlated with axial length (AL) even after correction.^11^^–^^13^ Our previous study^14^ showed that AL is inversely related to NFLT, NFLP-CD, and GCCT due to a combination of the optical magnification effect and anatomic variance, which agrees with prior literature.^15^^–^^22^ Notably, the superior vascular complex, a slab known to indicate macular perfusion loss, did not correlate with axial length^23^ and was not further investigated in this study. The refractive error–associated biases seen in NFLT, NFLP-CD, and GCCT were associated with increased false-positive rates. Fortunately, regression-based adjustments using AL or spherical equivalent (SE) refraction helped reduce this diagnostic bias. However, the quantitative OCT and OCTA measurements in the pilot study represented only the global averages of each scan parameter, which does not account for potential sectoral differences.

In the current study, we investigated the sectoral variance in the associations among NFLT, GCCT, and NFLP-CD and measures of axial ametropia: AL, SE manifest refraction, and apparent optic disk diameter (DD) measured on OCT. Similar to the findings from our prior study, we found elevated false-positive rates in the high and low myopia groups for individual sectors. We then decreased elevated false-positive rates in OCT/OCTA diagnostic parameters based on sector-specific adjustments.

## Methods

The participants in this study are from a subgroup of the Hong Kong FAMILY Cohort, a large population-based study with a territory-wide random sample.^24^ The study adhered to the tenets of the Declaration of Helsinki and was approved by the Institutional Review Board of the University of Hong Kong. The recruitment process and cohort characteristics were previously reported.^25^^,^^26^

The relevant ocular assessment for this study included AL measured with an ocular biometer (AL-Scan; NIDEK, Gamagori, Japan). The best possible correction based on subjective refraction provided the best-corrected visual acuity. Retinal imaging was obtained by a spectral-domain OCT device with OCTA function (Avanti with AngioVue Analytics 2017.1.0.151; Optovue, Fremont, CA).^26^ The NFLT was measured using the standard 3.4-mm-diameter circle from the structural optic nerve head OCT scan. Apparent disk size, as measured by the average diameter, is a potential surrogate measure for magnification. For that purpose, the magnification should be proportional to the actual average disk diameter calculated as 2 times the square root of the apparent optic disk area divided by π. The GCCT was based on 6 × 6-mm OCT scans of the macula shifted 1 mm temporal to the fovea. The NFLP-CD was based on 4.5 × 4.5-mm OCTA scans of the optic disk region. Sectoral measurements of NFLT and NFLP-CD were based on eight 45° sectors: temporal upper (TU), superior temporal (ST), superior nasal (SN), nasal upper (NU), nasal lower (NL), inferior nasal (IN), inferior temporal (IT), and temporal lower (TL). GCCT measurements were based on superior and inferior hemispheres.

This study included either one or two normal eyes from each participant. Individuals with the following were excluded: history of glaucoma, abnormalities in frequency doubling technology perimetry or fundus examination (disk, macula, or vessels), elevated intraocular pressure (>21 mmHg), enlarged cup-to-disk ratio (>0.7), and pseudophakia. Individuals with scans missing any OCT/OCTA data or measurements were excluded. Images with a low signal strength index (<50) were also excluded. Scans were manually evaluated for the presence of motion artifacts.

Multivariable broken stick regression analyses were performed for global and sectoral OCT and OCTA parameters. Age, sex, and signal strength index (SSI) were included in all regression models. In contrast, AL, SE, and DD were included in each model one at a time. Breakpoints of the broken stick models were set at emmetropia (SE = 0 diopters [D]). Based on our initial study, which showed that myopic and hyperopic eyes have differing associations with OCT and OCTA parameters, we continued to use multivariable broken stick linear regression models to separate the myopes and hyperopes in this follow-up study.^23^ The slopes of the myopic and hyperopic segments in the regression model were considered significantly different if *P* < 0.05 for the interaction term between the axial ametropia variable and a variable that dichotomizes myopia versus hyperopia. A linear mixed model was applied to the multivariable regression analysis to address the issue of within-participant clustering.

The adjustment of OCT parameters for age, sex, SSI, and AL was based on coefficients from the regression models using the following formula: Adjusted OCT or OCTA diagnostic parameter = (OCT or OCTA diagnostic parameter) – (age – 50) × slope coefficient [age] – sex × slope coefficient [sex] – (SSI – 50) × slope coefficient [SSI] – (AL – 23.7) × slope coefficient [AL]. The age reference value was the mean age of the emmetropia group, which was 50. The SSI reference set at 50 was the cutoff for a low-quality scan, as described in the exclusion criteria. The AL reference value was 23.7 mm, the mean AL from the emmetropia group.

A similar formula was used for SE: Adjusted OCT or OCTA diagnostic parameter = (OCT or OCTA diagnostic parameter) – (age – 50) × slope coefficient [age] – sex × slope coefficient [sex] – (SSI – 50) × slope coefficient [SSI] – SE × slope coefficient [SE].

A similar formula was also used for DD: Adjusted OCT or OCTA diagnostic parameter = (OCT or OCTA diagnostic parameter) – (age – 50) × slope coefficient [age] – sex × slope coefficient [sex] – (SSI - 50) × slope coefficient [SSI] – DD × slope coefficient [DD]. Global parameters were adjusted using the global parameter regression slope, whereas the sectors were adjusted using the individual sectoral regression slopes. These OCT parameters were subsequently labeled as AL-, SE-, or DD-adjusted after adjustment. Slopes standardized against the standard deviation of both dependent and independent variables were included to compare the effect sizes among covariates.

The eyes included in this study were stratified based on spherical equivalent: high myopia (<–6 D), low myopia (–6 D to –1 D), emmetropia (–1 D to 1 D), and hyperopia (>1 D) based on broad definitions reported in the literature.^27^^–^^30^ An “abnormal” classification threshold for each parameter (NFLT, GCCT, and NFLP) was set at the fifth percentile of the emmetropic eyes. Thus, eyes with OCT and OCTA diagnostic parameters below the threshold were deemed false positives because all eyes included in the study were considered normal. The false-positive rate, calculated by dividing the number of false-positive eyes over the total number of eyes in each group, described the refractive error–related bias. The χ^2 ^ test was used to determine the statistical significance of any difference in false-positive rates between groups (e.g., emmetropia vs. low myopia). The McNemar test was used to determine the statistical significance of any reduction in false-positive rates based on regression-based adjustments. An additional analysis of a random single-eye subset was also performed to ensure that any correlation between the eyes of a single individual did not significantly affect the results or conclusions.

Statistical analysis was conducted in R 4.0.2 (R Foundation for Statistical Computing, Vienna, Austria) using RStudio 1.3 (Posit, PBC, Boston, MA). Figures highlighting the false-positive rates were created using Prism 9.0.2 for Mac (GraphPad Software, San Diego, CA). To correct for multiple comparisons, a Bonferroni correction was used with a factor 8 to reflect the eight NFLT/NFLP sectors. As a result, *P* < 0.00625 denoted significance when comparing the slopes between the global and the individual sectors.

## Results

A total of 3936 eyes from 1968 participants were enrolled in the study. After applying the exclusion criteria, a total of 1339 eyes from 789 participants with a mean ± standard deviation (SD) of 46.9 ± 14.7 years (range, 18–78) were included (Table 1). All participants self-identified as Asian. Structural analysis of the peripapillary region revealed that the parameters (axial eye length and SE refractive error) that affect optical magnification introduced the largest bias compared to covariates in all sectors except temporally (Table 2). Even after standardization, the slopes of the temporal sectors were significantly different from that of the global parameter for the temporal sectors: TU, TL, and IT (ANOVA *F*-test with mixed effects; *P* < 0.001, using a Bonferroni correction). There was also variation among the raw regression slopes of the eight sectors. The superior, inferior, and nasal nerve fiber layer sectors were thinner in myopic eyes with increasing magnification (shorter AL and less myopic SE), whereas the temporal sectors (TU or TL) were thicker (Table 2).

**Table 1.** Demographic and Ocular Characteristics of the Study Participants

Parameter	All	High Myopia (<−6 D)	Low Myopia (−6 D to −1 D)	Emmetropia (−1 D to 1 D)	Hyperopia (>1 D)	*P*
Participants, *n*	789	66	336	284	103	—
Eyes, *n*	1339	117	590	480	152	—
Age (y)	46.9 ± 14.7	40.3 ± 11.3	42.0 ± 14.1	50.3 ± 13.9	58.2 ± 11.8	<0.001
Male sex, %	42	44	43	42	39	<0.001
IOP (mm Hg)	13.7 ± 2.7	14.0 ± 2.5	14.1 ± 2.8	13.3 ± 2.7	13.0 ± 2.6	<0.001
SE (D)	−1.8 ± 2.9	−7.9 ± 1.6	−3.1 ± 1.4	0.1 ± 0.6	2.0 ± 1.4	<0.001
AL (mm)	24.5 ± 1.4	26.7 ± 1.0	25.0 ± 0.9	23.7 ± 1.0	23.3 ± 1.1	<0.001
DD (mm)	1.60 ± 0.17	1.50 ± 0.18	1.56 ± 0.16	1.65 ± 0.16	1.68 ± 0.16	<0.001
NFLT (µm)	100.6 ± 9.0	95.2 ± 8.4	99.2 ± 9.0	102.9 ± 8.2	102.9 ± 9.5	<0.001
NFLT SSI	66.5 ± 8.0	64.6 ± 7.4	67.3 ± 7.6	66.8 ± 8.2	64.0 ± 8.5	<0.001
NFLP-CD area (%)	54.1 ± 2.8	53.0 ± 3.0	54.0 ± 2.8	54.7 ± 2.7	54.0 ± 2.7	<0.001
NFLP-CD SSI	66.8 ± 7.7	63.1 ± 6.2	66.6 ± 7.3	68.0 ± 8.0	65.9 ± 8.1	<0.001
GCCT (µm)	96.3 ± 6.8	94.6 ± 7.4	95.4 ± 6.3	97.5 ± 6.8	97.4 ± 7.6	<0.001
GCCT SSI	71.8 ± 7.1	68.3 ± 5.5	71.3 ± 6.8	73.3 ± 7.2	71.4 ± 7.8	<0.001

The study participants were stratified by spherical equivalent (SE), and the group statistics are displayed as mean ± SD. The difference among groups of continuous and categorical (e.g., sex) variables was tested by generalized linear mixed models to address within-participant clustering. Age was tested by analysis of variance. SSI is an integer score from 0 to 100 based on the reflectance signal. AL, axial length; CD, capillary density; DD, disk diameter; GCCT, ganglion cell complex thickness; IOP, intraocular pressure; NFLP, nerve fiber layer plexus; NFLT, nerve fiber layer thickness; SE, spherical equivalent refraction; SSI, signal strength index; SVC, superficial vascular complex; VD, vessel density.

**Table 2.** Multivariable Linear Regression Analyses of the NFLT Sectors

	NFLT Sector (µm)								
	Global	TU	ST	SN	NU	NL	IN	IT	TL
**Model 1**									
Age (y)	−0.14 (−0.22)***	−0.15 (−0.24)***	−0.26 (−0.42)***	−0.16 (−0.26)***	−0.01 (−0.02)	−0.01 (−0.02)	−0.11 (−0.18)**	−0.33 (−0.52)***	−0.10 (−0.15)***
Male sex (%)	−0.82 (−0.04)	−4.44 (−0.24)***	1.94 (0.11)	1.88 (0.10)	1.35 (0.07)	−0.12 (−0.01)	−1.32 (−0.07)	−3.97 (−0.22)***	−1.27 (−0.07)
SSI	0.09 (0.08)***	−0.01 (−0.01)	0.13 (0.12)***	0.13 (0.12)**	0.09 (0.08)*	0.02 (0.02)	0.09 (0.08)	0.14 (0.12)**	0.03 (0.03)
AL (mm)	−2.22 (−0.34)***	0.53 (0.08)	−2.86 (−0.44)***	−4.31 (−0.66)***	−3.25 (−0.50)***	−2.59 (−0.40)***	−5.55 (−0.85)***	−1.27 (−0.20)**	0.84 (0.13)**
**Model 2**									
Age (y)	−0.14 (−0.22)***	−0.16 (−0.25)***	−0.29 (−0.46)***	−0.16 (−0.26)***	−0.01 (−0.01)	−0.01 (−0.01)	−0.10 (−0.16)*	−0.35 (−0.55)***	−0.10 (−0.16)***
Male sex (%)	−1.89 (−0.10)**	−4.21 (−0.23)***	0.59 (0.03)	−0.14 (−0.01)*	−0.21 (−0.01)	−1.35 (−0.07)	−3.97 (−0.22)***	−4.59 (−0.25)***	−0.88 (−0.05)
SSI	0.09 (0.08)***	−0.01 (−0.01)***	0.14 (0.12)**	0.15 (0.13)**	0.10 (0.09)*	0.03 (0.03)	0.11 (0.10)*	0.14 (0.13)**	0.03 (0.02)
SE (D)	1.02 (0.33)***	−0.28 (−0.09)	1.62 (0.51)***	1.84 (0.59)***	1.61 (0.51)***	1.28 (0.41)***	2.37 (0.75)***	0.95 (0.30)***	−0.36 (−0.11)**
**Model 3**									
Age (y)	−0.11 (−0.17)***	−0.16 (−0.26)***	−0.23 (−0.36)***	−0.10 (−0.17)**	0.03 (0.04)	0.02 (0.03)	−0.04 (−0.07)	−0.31 (−0.50)***	−0.11 (−0.17)***
Male sex (%)	−1.77 (−0.10)**	−4.21 (−0.23)***	0.73 (0.04)	0.07 (0.01)	0.01 (0.01)	−1.16 (−0.06)	−3.67 (−0.20)**	−4.51 (−0.25)***	−0.92 (−0.05)
SSI	0.09 (0.08)***	−0.02 (−0.02)	0.15 (0.13)**	0.16 (0.14)**	0.11 (0.10)**	0.04 (0.04)	0.12 (0.10)*	0.15 (0.13)**	0.02 (0.02)
DD (mm)	16.16 (0.15)***	−4.85 (−0.05)***	27.46 (0.26)***	46.19 (0.43)***	45.19 (0.43)***	39.69 (0.37)***	45.56 (0.43)***	8.87 (0.08)	−9.49 (−0.09)**

All broken stick regression models included age, sex, and SSI with the addition of AL (model 1), SE (model 2), or DD (model 3) separately in each model. The dependent variables are the column headings in thickness (µm). The data are displayed as the slope followed by the parentheses representing the slope standardized against the standard deviations (SDs) of both dependent and independent variables. Statistical significance is as follows: ***P < 0.001, **P < 0.01, *P < 0.05. The standardized slope is the change in SD of the OCT parameter per 1 SD of the independent variable. Only the slopes for the myopic segments are shown; the slopes for the hyperopic segments were not statistically significant. AL, axial length; DD, disk diameter; IN, inferior nasal; IT, inferior temporal; NFLT, nerve fiver layer thickness; NL, nasal lower; NU, nasal upper; SE, spherical equivalent; SN, superior nasal; SSI, signal strength index; ST, superior temporal; TL, temporal lower; TU, temporal upper.

In the macular region, AL and SE introduced the greatest magnification bias in both hemispheres compared to the other covariates (Table 3). The global and sectoral GCCT slopes were not significantly different (ANOVA *F*-test with mixed effects; *P* = 0.43) (Table 3). However, raw regression slope variations were still apparent among the global and sectoral parameters, as well as among individual sectors.

**Table 3.** Multivariable Linear Regression Analyses of the GCCT Hemispheres

	GCCT Global (µm)	GCCT Superior (µm)	GCCT Inferior (µm)
**Model 1**			
Age (y)	−0.05 (−0.10)**	−0.05 (−0.10)**	−0.06 (−0.11)**
Male sex (%)	0.46 (0.03)	0.21 (0.01)	0.74 (0.05)
SSI	0.06 (0.06)**	0.06 (0.06)**	0.06 (0.06)*
AL (mm)	−0.91 (−0.17)***	−0.78 (−0.15)***	−1.04 (−0.20)***
**Model 2**			
Age (y)	−0.05 (−0.10)**	−0.04 (−0.09)**	−0.05 (−0.10)**
Male sex (%)	0.03 (0.00)	−0.16 (−0.01)	0.24 (0.02)
SSI	0.07 (0.06)**	0.07 (0.07)**	0.06 (0.06)**
SE (D)	0.38 (0.15)***	0.35 (0.14)***	0.43 (0.17)***
**Model 3**			
Age (y)	−0.04 (−0.08)*	−0.04 (−0.07)*	−0.04 (−0.08)*
Male sex (%)	0.08 (0.01)	−0.11 (−0.01)	0.30 (0.02)
SSI	0.07 (0.07)**	0.07 (0.07)**	0.07 (0.07)**
DD (mm)	8.38 (0.10)***	8.03 (0.09)***	9.61 (0.11)***

All broken stick regression models included age, sex, and SSI with the addition of AL (model 1), SE (model 2), or DD (model 3) separately in each model. The dependent variables are the column headings. Data are displayed as the slope (slope standardized against the SDs of both dependent and independent variables) and statistical significance: ***P < 0.001, **P < 0.01, *P < 0.05. The standardized slope is the change in SD of the OCT parameter per 1 SD of the independent variable. Only the slopes for the myopic segments are shown; the slopes for the hyperopic segments were not statistically significant. AL, axial length; CD, capillary density; DD, disk diameter; SE, spherical equivalent; SSI, signal strength index.

**Figure 1.**
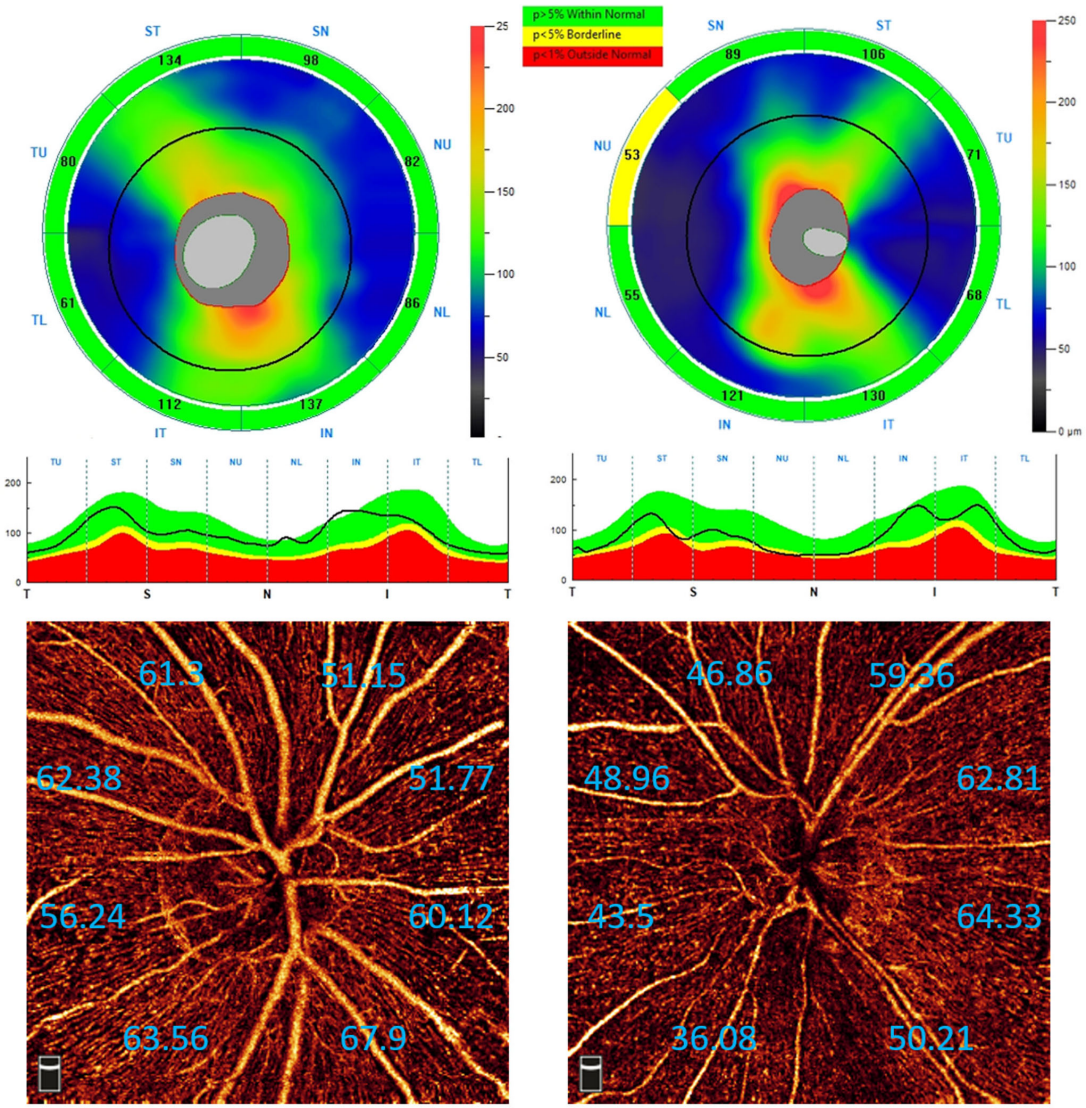
A comparison between an emmetropic (SE = 0 D) right eye and a highly myopic (SE = −9 D) left eye. Both eyes were selected around the 25th percentile of the overall nerve fiber layer (NFL) thickness in each refractive error group. The top row shows peripapillary NFL thickness maps (4.9 mm) and eight sectoral averages of 45° sectors. Please note that the sectors are mirrored in the horizontal direction in the left eye. The middle row shows NFL thickness profile at circle with 3.4-mm diameter, both following a temporal–superior–nasal–inferior–temporal (TSNIT) direction, and the bottom row shows corresponding peripapillary NFL plexus angiograms (4.5 mm) and eight sectoral average of NFL plexus capillary density (NFLP-CD). Each sector is 45°, and the sectors are mirrored in the horizontal direction in the left eye.

Angiographic analysis of the peripapillary region revealed that the magnification bias had a smaller impact than SSI (Table 4). Among the eight sectors for the AL model, the slopes of three temporal sectors (TL, TU, and IT) significantly differed from that of the global NFLP-CD (ANOVA *F*-test with mixed effects; *P* < 0.006, using a Bonferroni correction). These three sectors also had relatively lower residual variance (12.65–13.71 vs. 19.42–23.99) (Supplementary Table S1). In general, a greater global NFLP-CD was significantly associated with increasing magnification except in the temporal sectors. DD was not associated with NFLP globally, but larger DD was correlated with higher NFLP-CD in all except two inferior sectors. To illustrate the difference of overall and local differences in the peripapillary area, we compared the OCT/OCTA of an emmetropic eye and a high myopia eye (Fig. 1). Both eyes were selected from the 25th percentile of the overall NFL thickness of each refractive error group. Overall, both the NFL thickness and NFLP-CD were lower in the high myopia eye. The trend was the same for superior, inferior, and nasal sectors, but the temporal sectors of high myopia eyes had a higher value.

**Table 4.** Multivariable Linear Regression Analyses of the NFLP Sectors

	NFLP Sector								
	Global	TU	ST	SN	NU	NL	IN	IT	TL
**Model 1**									
Age (y)	−0.02 (−0.08)***	−0.02 (−0.07)*	−0.01 (−0.04)	−0.01 (−0.02)	0.05 (−0.16)***	0.03 (0.10)*	0.01 (0.03)	0.02 (0.05)	−0.06 (−0.18)***
Males sex (%)	−0.89 (−0.10)***	−1.25 (−0.13)***	−0.09 (−0.01)	−0.58 (−0.06)	−0.44 (−0.05)	−0.54 (−0.06)	−1.57 (−0.17)***	−1.42 (−0.15)***	−0.39 (−0.04)
SSI	0.17 (0.28)***	0.14 (0.23)***	0.09 (0.16)***	0.13 (0.22)***	0.22 (0.37)***	0.23 (0.39)***	0.13 (0.22)***	0.14 (0.23)***	0.17 (0.29)***
AL (mm)	−0.32 (−0.10)***	0.11 (0.03)	−0.52 (−0.16)***	−0.30 (−0.09)*	−0.39 (−0.12)**	−0.56 (−0.17)***	−0.63 (−0.19)***	−0.13 (−0.04)	−0.07 (−0.02)
**Model 2**									
Age (y)	−0.02 (−0.08)***	−0.02 (−0.08)*	−0.02 (−0.05)	−0.01 (−0.02)	0.05 (0.16)***	0.03 (0.10)*	0.01 (0.03)	0.01 (0.04)	−0.06 (−0.19)***
Male sex (%)	−1.04 (−0.11)***	−1.20 (−0.13)***	−0.33 (−0.05)	−0.71 (−0.08)*	−0.61 (−0.07)	−0.81 (−0.09)*	−1.86 (−0.20)***	−1.47 (−0.16)***	−0.34 (−0.04)
SSI	0.17 (0.29)***	0.13 (0.22)***	0.09 (0.15)***	0.13 (0.22)***	0.22 (0.37)***	0.23 (0.39)***	0.13 (0.22)***	0.13 (0.22)***	0.16 (0.27)***
SE (D)	0.14 (0.09)***	0.07 (0.05)	0.40 (0.25)***	0.11 (0.07)	0.17 (0.11)*	0.37 (0.23)***	0.35 (0.22)***	0.12 (0.07)*	0.07 (0.04)
**Model 3**									
Age (y)	−0.02 (−0.06)***	−0.02 (−0.08)*	−0.01 (−0.02)	0.00 (0.00)	0.06 (0.17)***	0.04 (0.12)**	0.02 (0.06)	0.02 (0.06)*	−0.06 (−0.19)***
Male sex (%)	−1.03 (−0.11)***	−1.18 (−0.13)***	−0.31 (−0.03)	−0.71 (−0.08)*	−0.58 (−0.06)	−0.75 (−0.08)*	−1.84 (−0.20)***	−1.47 (−0.16)***	−0.34 (−0.04)
SSI	0.18 (0.29)***	0.13 (0.22)***	0.10 (0.17)***	0.14 (0.22)***	0.22 (0.37)***	0.24 (0.39)***	0.14 (0.24)***	0.14 (0.23)***	0.16 (0.27)***
DD (mm)	1.03 (0.02)	7.94 (0.15)***	9.52 (0.18)***	3.77 (0.07)*	13.42 (0.25)***	15.16 (0.28)***	3.21 (0.06)	2.84 (0.05)	6.03 (0.11)***

All broken stick regression models included age, sex, and SSI with the addition of AL (model 1), SE (model 2), or DD (model 3) separately in each model. The dependent variables are the column headings in capillary density (% area). Data are displayed as the slope (slope standardized against the SDs of both dependent and independent variables) and statistical significance: ***P < 0.001, **P < 0.01, *P < 0.05. The standardized slope is the change in standard deviation (SD) of the OCT parameter per 1 SD of the independent variable. Only the slopes for the myopic segments are shown; the slopes for the hyperopic segments were not statistically significant. AL, axial length; DD, disk diameter; IN, inferior nasal; IT, inferior temporal; NFLP, nerve fiber layer plexus, NL, nasal lower; NU, nasal upper; SE, spherical equivalent; SN, superior nasal; SSI, signal strength index; ST, superior temporal; TL, temporal lower; TU, temporal upper.

**Figure 2.**
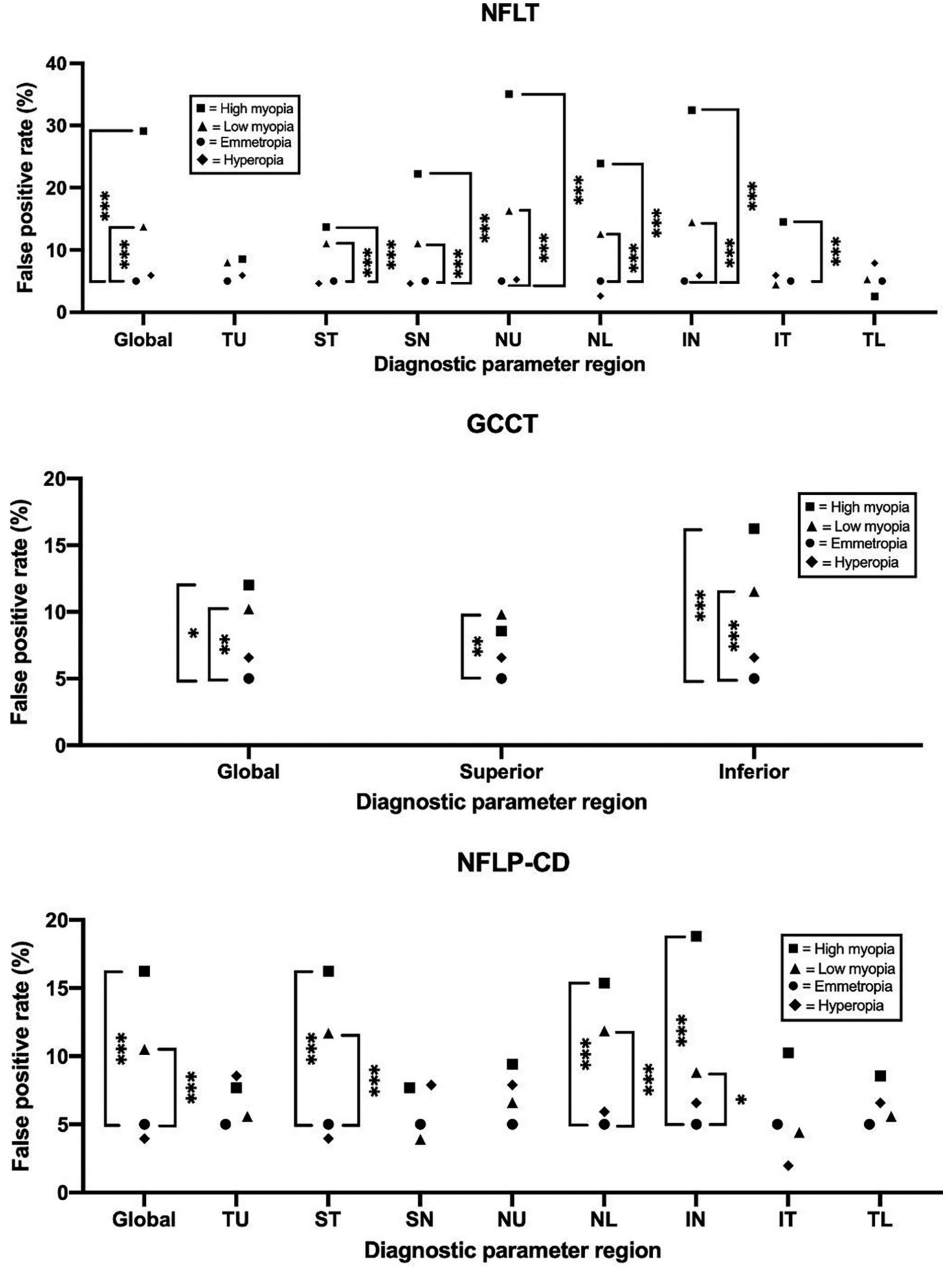
False-positive rates of global and sectoral glaucoma diagnostic parameters are stratified by refractive error based on shape. Diagnostic parameters were adjusted using multivariable regression against age, sex, and signal strength index. Brackets between points indicate statistically significant differences from χ^2^ tests: ***P < 0.001, **P < 0.01, and *P < 0.05. There was no statistical significance between hyperopia and emmetropia in NFLT, NFLP-CD, or GCCT. GCCT, ganglion cell complex thickness; IN, inferior nasal; IT, inferior temporal; NFLP-CD, nerve fiber layer plexus capillary density; NFLT, nerve fiber layer thickness; NL, nasal lower; NU, nasal upper; SN, superior nasal; ST, superior temporal; TL, temporal lower; TU, temporal upper.

Compared to the false-positive rates of the emmetropic group, the false-positive rates in the high and low myopia groups were significantly (χ^2^^ ^test, *P* < 0.001) elevated for the global NFLT, NFLP-CD, and GCCT values (Fig. 2). No significant difference was found in false-positive rates between the emmetropic and hyperopic groups, which agrees with the trends in diagnostic parameters among subgroups and supports the need for broken stick regression models to fit refractive error–associated biases better. Sector analysis showed that all sectors of NFLT except for the temporal sectors demonstrated that greater AL was associated with a significant increase in false-positive rates. In GCCT, only the false-positive rate in the low myopia group for the superior hemisphere differed significantly from that of the emmetropic group (Fig. 2, middle). Both low and high myopia eyes agreed with the global GCCT trends in the inferior hemisphere. Finally, in NFLP-CD, only the ST, NL, and IN sectors agreed with the global NFLP-CD trends (Fig. 2, bottom). In NFLT, GCCT, and NFLP-CD, individual sectors had a greater false-positive rate than the global parameter—indicating the need for a sector-specific adjustment.

**Figure 3.**
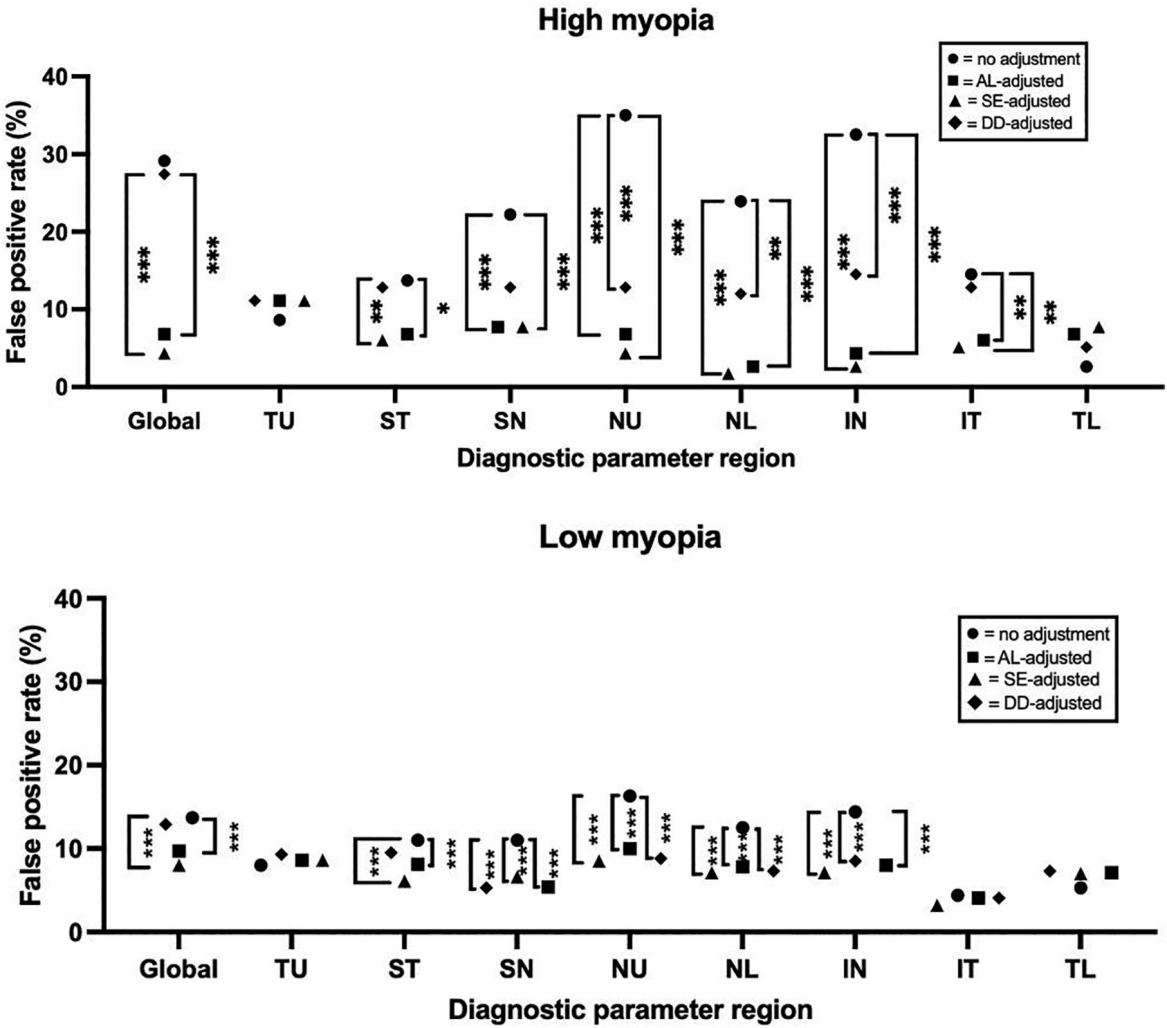
False-positive rates of the NFL thickness stratified by adjustment type based on shape. Brackets between points indicate statistically significant differences from McNemar tests: ***P < 0.001, **P < 0.01, *P < 0.05. AL, axial length; DD, disk diameter; NFL, nerve fiber layer; SE, spherical equivalent.

**Figure 4.**
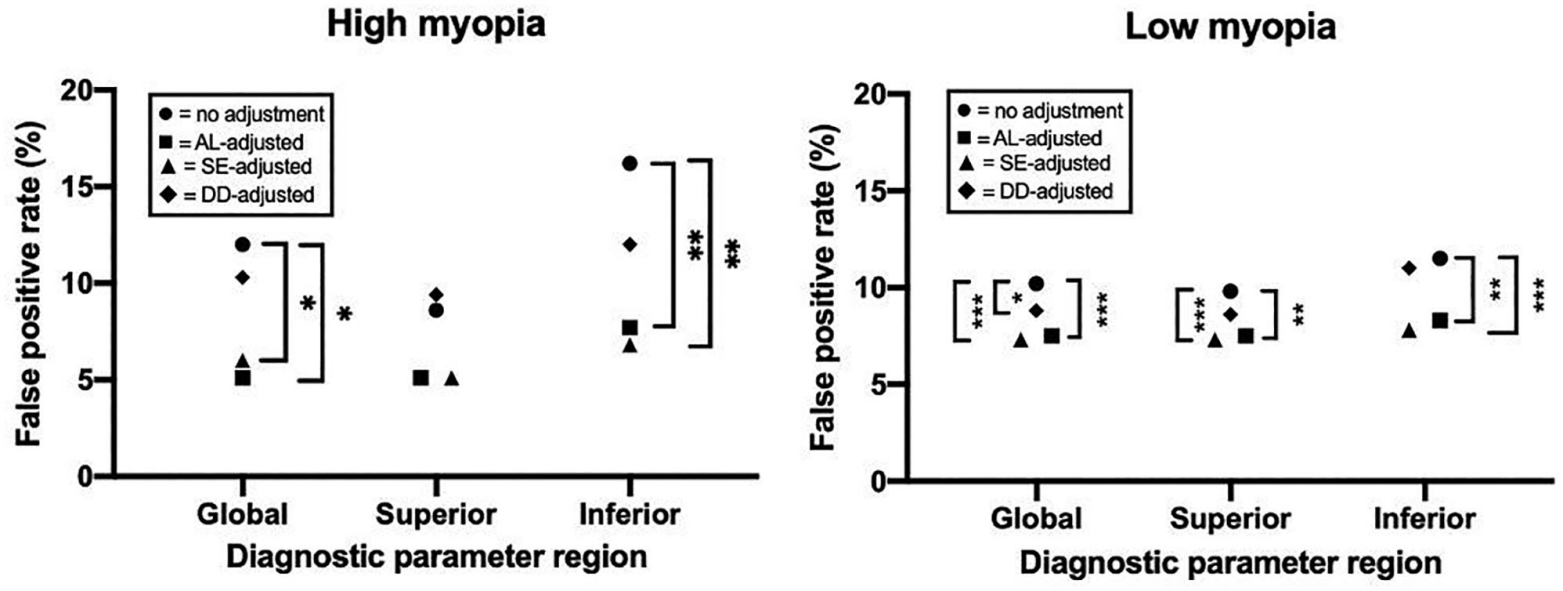
False-positive rates of the ganglion cell complex thickness stratified by adjustment type based on shape. Brackets between points indicate statistically significant differences from McNemar tests: ***P < 0.001, **P < 0.01, *P < 0.05. AL, axial length; DD, disk diameter; SE, spherical equivalent.

**Figure 5.**
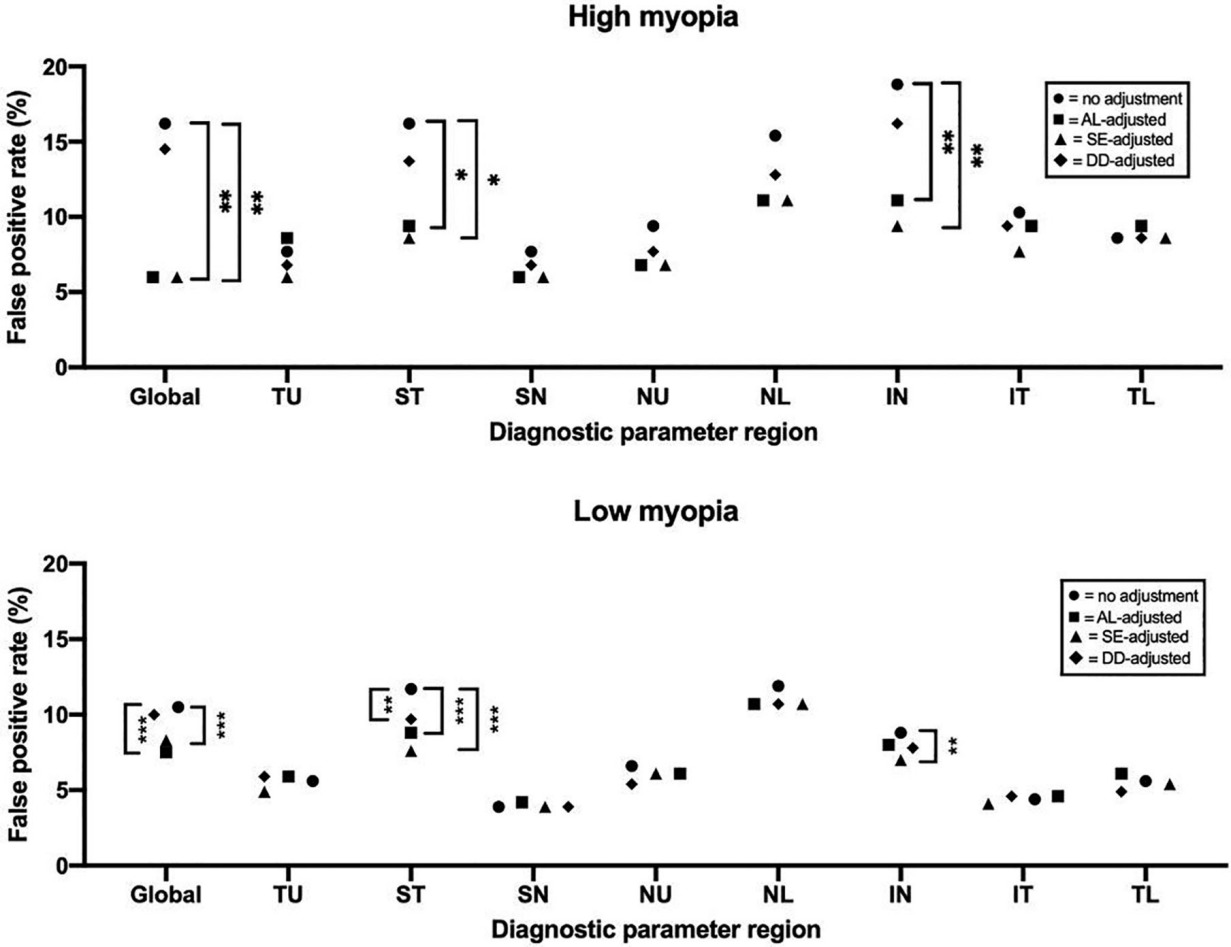
False-positive rates of the NFL plexus capillary density stratified by adjustment type based on shape. Brackets between points indicate statistically significant differences from McNemar tests: ***P < 0.001, **P < 0.01, *P < 0.05. AL, axial length; DD, disk diameter; IN, inferior nasal; IT, inferior temporal; NFL, nerve fiber layer; NL, nasal lower; NU, nasal upper; SE, spherical equivalent; SN, superior nasal; ST, superior temporal; TL, temporal lower; TU, temporal upper.

Regression-based adjustments with AL and SE from individual sectoral parameters significantly reduced the false-positive rates in the high and low myopia groups for global NFLT and most sectors except temporally (Fig. 3). In GCCT, AL- and SE-based adjustments helped reduce the false-positive rate globally and in the inferior hemisphere but not in the superior hemisphere (Fig. 4). In NFLP, AL- and SE-based adjustments were only significantly associated with lower false-positive rates for the global, IN, and ST sectors (Fig. 5). Finally, DD adjustment did not significantly reduce the false-positive rate for any global or regional diagnostic parameter, even when sector-specific adjustments were utilized.

In addition to AL, SE, and DD, other significant covariates included in the regression models were SSI, age, and sex. Higher SSI was significantly associated with increased NFLT in the superior and inferior sectors, superior and inferior GCCT hemispheres, and all NFLP-CD sectors. Notably, the SSI was a more impactful covariate than AL and SE in the NFLP-CD models, whereas the opposite was true in the NFLT and GCCT models. Increasing age was associated with significantly reduced NFLT in all sectors except nasally, decreased GCCT in both hemispheres, and lower NFLP-CD in the temporal and nasal sectors. Male sex was significantly correlated with reduced NFLT in the temporal and inferior sectors and lower NFLP-CD in the inferior and temporal upper sectors. Sex was not associated with GCCT.

## Discussion

In this large community-based study, we found that the effect of magnification variance is even more impactful in sector-based glaucoma diagnostic parameters than in global parameters. The nasal NFLT sectors showed an extremely high false-positive rate in myopes, at times even more than the global NFLT parameter. A similar pattern is seen in certain hemispheres of GCCT and sectors in NFLP-CD. Thus, an adjustment based on the individual sector of each parameter is more appropriate than one based on the global parameter considering the sectoral variance within each glaucoma diagnostic parameter.

The superior, nasal, and inferior sectors of the NFLT all agreed with the global NFLT, where increasing AL was associated with a thinner overall nerve fiber layer. However, in the temporal sectors of myopes, an increased AL correlated with a thicker NFLT, which agrees with prior studies.^11^^,^^15^ In myopes, the nerve fiber layer scan area and the diameter of the sampling circle are widened in proportion to axial myopia due to a reduction in the optical magnification, as described previously by our group.^19^^,^^23^ The apparent temporal shift may be due to the arcuate course of nerve fibers, which turns temporally at larger radial distance from the disk center.^11^^,^^31^^–^^34^ The difference between the temporal sectors and the other sectors for NFLT was further supported in the standardized slope analysis, where the temporal sectors were the only ones with a significantly different slope than the global parameter. The difference among sectors also translated over to the false-positive rate analysis. The global NFLT parameter and the superior, nasal, and inferior sectors all demonstrated increased false-positive rates in myopic eyes, but the temporal sectors remained unaffected. Therefore, sector-based adjustment for NFLT is needed because magnification variance affects the sectors differently.

For peripapillary NFLP-CD, an increased false-positive rate was associated with increased AL globally and in several sectors. The pattern in NFLP-CD is similar to NFLT as they are highly correlated. However, the effect on NFLP-CD may be less pronounced because capillary density decreases more slowly as a function of distance from the optic disk.^22^ Still, sector-based adjustment for NFLP-CD is also needed because magnification variance affects the sectors differently.

In the macular region, the inferior GCCT hemisphere showed a stronger association with axial ametropia measures than the superior GCCT hemisphere (Table 3), which agrees with previous studies.^17^^,^^35^ Because the fovea is inferiorly displaced relative to the disk, the superior and inferior nerve fiber bundles do not have symmetric trajectories.^32^ The superior bundles run nearly parallel to the upper edge of the macular scan, whereas the inferior bundles fan out more rapidly and with an inferotemporal slant at the bottom boundary of the macular scan. This explains why the measurements in the inferior macula are affected more severely by the magnification variance.

Based on the broken stick regression models applied to the NFLT sectors, the increased false-positive rates of the superior, nasal, and inferior sectors can be reduced with either AL or SE-based adjustments from specific sectors. For NFLP, most sectors with increased false-positive rates benefited from AL- and SE-based adjustments to reduce bias. The sectors without significant improvement from adjustments mainly were ones where the false-positive rates, even without adjustment, were only 5% to 10%. Most sectors still showed a trend of improvement from AL- and SE-based adjustments—especially in the high myopia group. In GCCT, regression-based adjustments using AL and SE reduced the increased GCCT false-positive rates in myopic eyes.

Aside from AL and SE, other covariates (age, sex, and SSI) should also warrant adjustment. Adjustment of SSI is critical in NFLP-CD, where SSI has the greatest effect size compared to all other variables. To determine flow, the OCTA machine evaluates the change in reflectance of sequential OCT images. Many studies have shown the correlation between quantitative parameters and OCT/OCTA reflectance signal strength.^36^^–^^38^ Thus, accounting for the SSI is essential in minimizing potential bias in angiographic parameters. Adjustments of age, sex, and SSI are also important in GCCT and NFLT—especially in the temporal sectors where the associations of the imaging parameter with AL and SE are not as strong.

In our previous study, DD-based adjustment did not help reduce the elevated false-positive rates. This study shows that, even among the various sectors where DD had a comparable effect size to age or SSI (Tables 2^3^–4), adjustment for DD did not improve false-positive rates for NFLT, GCCT, and NFLP-CD parameters (Figs. 2^3^–4). The apparent disk diameter is affected by the actual disk diameter and may not be a good surrogate measure of transverse optical magnification. This explains why it was not helpful in the regression-based adjustment of OCT/OCTA diagnostic parameters.^39^

In this study, we used linear regression for magnification adjustment because the formula can be directly used on data from the clinical report. Alternatively, other methods, such as resampling on the scan area^32^^,^^40^^,^^41^ or deep learning,^42^^,^^43^ may also work.

A clarification is needed regarding our choice of classification threshold for abnormality, which is based on the 5th-percentile cutoff in emmetropic eyes. This maximizes our ability to contrast false-positive rates between emmetropes and myopes but can differ from actual clinical practice. In common practice, structural and perfusion values are separately flagged if they are below the 1st-percentile cutoff or between the 1st- and ∼5th-percentile cutoffs. By simplifying this to a single 5th-percentile cutoff, we reduced the number of statistical comparisons, which was helpful as we were already making parallel comparisons of multiple sectors. In this study, the classification threshold is based on a reference group of near-emmetropes with 1 D or less of myopia or hyperopia. This more narrowly defined reference group may have a moderately raised both true and false-positive rates than the typical manufacturer-provided normative database. But, if we had chosen to include myopes and hyperopes in the reference group, the threshold would be too loose, as our study population contained a higher proportion of myopes than a U.S. Food and Drug Administration reference population, which typically has a narrow distribution around emmetropia. We chose the narrower emmetropia range for conceptual clarity to contrast with the various types of ammetropes. A side benefit of the more stringent standard for normality may be higher rates of false positives in all groups, which would effectively increase the sample sizes and statistical power for comparison of emmetropes with both low and high myopes.

Several limitations are present in this study. The generalizability of our conclusions is limited to one race and one machine model. Future studies investigating the effect of magnification from measures of axial ametropia in other races and with different machine models and algorithms may be helpful. Additionally, this dataset is based on only normal, healthy eyes following strict exclusion criteria to remove pseudophakic eyes and ones with retinal or glaucoma-related pathologies. This study helps establish a modified baseline so that glaucoma damage might be more accurately detected in myopic patients, but it does not address the patterns of glaucoma damage in myopes, which may differ from those in emmetropes. This study included one or both eyes from some participants, which may contribute to the between-eye correlation. To address clustering within participants, a linear mixed model was applied to the multivariable regression analysis, and additional analyses of the χ^2^ and McNemar tests were also performed on a random single-eye sample to ensure that the conclusions were not different.

This large population-based study showcased the regional differences between OCT and OCTA parameters and their associations with axial length, spherical equivalent refraction, and optic disk diameter. The effect of optical magnification was more impactful on certain sectors than the global parameter—as seen in the raw regression slopes and the elevated false-positive rates. Sector-specific adjustments based on AL and SE successfully reduced these biases. We hope this study sheds light on the sectoral variance of commonly used glaucoma diagnostic parameters, provides a feasible solution to addressing refractive error–related biases, and ultimately informs future clinical decision-making.

## Supplementary Material

Supplement 1
