# Supplementary material for: Sector-Based Regression Strategies to Reduce Refractive Error-Associated Glaucoma Diagnostic Bias When Using OCT and OCT Angiography
Source: Transl Vis Sci Technol. 2023 Sep 15;12(9):10. doi: 10.1167/tvst.12.9.10 (PMC10506684; doi:10.1167/tvst.12.9.10)
Supplement: Supplement 1 [file tvst-12-9-10_s001.pdf]

Supplemental Table 1: Variance of the nerve fiber layer thickness and plexus capillary density

	NFLT		NFLP-CD	
Sector	Residual variance	Conditional R^2	Residual variance	Conditional R^2
TU	62.1	0.61	12.7	0.39
ST	90.4	0.66	19.5	0.22
SN	105.3	0.64	19.4	0.34
NU	82.8	0.53	20.4	0.39
NL	48.0	0.63	24.0	0.46
IN	90.9	0.70	20.4	0.35
IT	95.1	0.70	12.9	0.39
TL	49.5	0.53	13.7	0.43

Mixed-effects intercept-only regression was conducted for each OCT metric to obtain residual variance and R^2. IN, inferior nasal; IT, inferior temporal; NFLT: nerve fiber layer thickness; NFLP-CD nerve fiber layer plexus capillary density; NL, nasal lower; NU, nasal upper; SN, superior nasal; ST, superior temporal; TL, temporal lower; TU, temporal upper.
